# Hypoxia silences the neural activities in the early phase of the phrenic neurogram of eupnea in the piglet

**DOI:** 10.1186/1743-0003-2-32

**Published:** 2005-11-30

**Authors:** Metin Akay

**Affiliations:** 1 Neural Engineering & Informatics Laboratory, Harrington Department of Bioengineering, Ira A. Fulton School of Engineering, Arizona State University, Tempe, AZ 85287-9709, USA

## Abstract

**Objective:**

We investigated phrenic neurogram patterns during eupnea (normal breathing) and severe hypoxia (gasping) during early maturation in the piglet.

**Methods:**

We used continuous wavelet transform and short time Fourier transform methods to examine the similarity of breathing patterns in both time and frequency domains during early maturation. The phrenic neurogram was recorded during eupnea, severe hypoxia, and recovery from severe hypoxia in piglets in three different age groups: 3–6 days, 10–15 days and 29–35 days.

**Results:**

During the first week of postnatal age, respiratory patterns of phrenic activity were marked by frequency components between 30 and 300 Hz during both the early (first half) and late (second half) phases of the neurogram signals during eupnea. The results suggest that there is little difference between the respiratory patterns in both time and frequency domains during eupnea compared to gasping for the first week of postnatal age in piglets. After the first week of postnatal age, the duration of the phrenic neurogram burst significantly increases and the patterns during the early phase of the phrenic neurogram are different from those observed for gasping. However, the patterns that mark the late phase of the phrenic neurograms are still the same as those of gasping.

**Conclusion:**

Our most significant finding is that hypoxia silences the neural activity in the early phase of phrenic neurogram regardless of maturation.

## Introduction

Production of progressive brain hypoxia in an anesthetized, vagotomized, peripherally-chemodenervated cat results in depression of respiratory output and a stereotypical progression of respiratory pattern changes, as hypoxia progresses [1-4]. Initially, the amplitude of the phrenic neurogram is depressed, with a fall in phrenic firing frequency only occurring as the hypoxia becomes more severe. As arterial O_2 _content falls, progressive respiratory depression continues until the phrenic output is completely silenced. If hypoxia is allowed to progress beyond this point, gasping will eventually ensue [6-8]. This form of respiration is characterized by brief, intense inspiratory efforts of the diaphragm and other respiratory muscles, and has been interpreted as an attempt at "autoresuscitation" [9-12]. This interpretation is based on the observation that animals asphyxiated to the point of apnea by airway occlusion, will restore arterial oxygenation quickly if the occlusion is removed and gasping ensues. If the animal fails to gasp, arterial oxygenation does not improve and death occurs due to cardiovascular collapse inevitably occurs.

The relationship of the medullary gasp to eupneic breathing has been a point of contention for a number of years. Lumsden originally conceived gasping as being the product of a primitive medullary pattern generator which does not contribute to eupneic breathing [6,13]. More recently, St. John and associates have, over the course of several studies, closely examined this question and have concluded that gasping is the result of a unique medullary pattern generator [14-16] in agreement with Lumsden's finding. This conclusion was based on studies of gasping produced by reversibly cooling the pontomedullary junction of decerebrate cats. Although the gasping produced by this procedure has timing characteristics which differ slightly from those seen during hypoxic gasping (e.g., shorter inspiratory time) [15], the qualitative changes seen in the phrenic neurogram and other respiratory outputs during gasping following cooling, were the same as those seen during hypoxic or asphyxic gasping [15,16]. With this model, it was first shown that gasping differs fundamentally from eupnea, both in the pattern of assembly of single phrenic motoneurons to produce a phrenic burst, and in timing characteristics of the phrenic neurogram. The central respiratory controller was also shown to be unresponsive to peripheral chemoreceptor stimulation during gasping. When gasping is produced in the decerebrate cat under conditions of carbon monoxide hypoxia, the discharge frequency of expiratory neurons falls sharply with some units becoming totally silent. The discharge frequency of inspiratory neurons is unchanged during gasping but, unlike during eupnea, all inspiratory neurons fire simultaneously at the beginning of the inspiratory period during gasping [17].

Respiratory control has been studied largely on the basis of phenomenology. There have also been attempts to apply empirical, analytical techniques to the study of central respiratory patterning. Cohen [18] was the first to use autospectral analysis of the phrenic neurogram to gain insight into the central respiratory pattern generation. Subsequently, numerous frequency domain analyses of the phrenic neurogram during eupnea, and during manipulations of various respiratory afferents, have been performed. Virtually all respiratory outputs studied during eupnea (e.g., phrenic and laryngeal neurograms; diaphragmatic electromyograms) have been shown to display two prominent peaks in their spectra: a medium-frequency oscillation (MFO) in the frequency range of 20–50 Hz, and a high-frequency oscillation (HFO) between 50–100 Hz [19-21]. A HFO spectral peak, which is correlated to the phrenic neurogram HFO, has also been noted in medullary inspiratory neuronal activity. Based on these observations, the HFO has been considered to be a characteristic of the central, respiratory pattern generator. The source of the MFO is more problematic [22]. Richardson and Mitchell [23] have proposed that the MFO arises from the interaction of two pattern generators, while Christakos et al. [24], interpret the MFO as a reflection of the rhythmic augmenting discharge of individual phrenic motorneurons resulting from an augmenting drive of supraspinal origin.

Richardson and Mitchell [24] compared the frequency spectra of the phrenic neurogram during eupnea and gasping in decerebrate cats. Hypoxic gasping in decerebrate cats was associated with a high-frequency peak in the phrenic neurogram at 120 Hz, as opposed to the 80 Hz peak seen during eupnea. Spectral analysis of occasional eupneic, phrenic bursts which showed gasp-like augmentation at the end of inspiration, revealed the presence of both eupneic and gasping high-frequency peaks. The presence of a unique spectral peak during gasping was presented as support for the idea that respiratory pattern generation differs during eupnea and gasping.

Preliminary studies of Akay et al. [25] used the modified Yule-Walker autoregression (AR) technique of spectral analysis to analyze 19 eupneic and 13 gasping, phrenic neurograms in anesthetized cats before and during CO-hypoxia and hypoxic-hypoxia, in two preliminary experiments. Our results suggested that eupnea is characterized by three peaks in the AR spectrum, with the lowest peak frequency between 30 and 60 Hz. During gasping a distinctive low-frequency peak was evident in the spectrum below 30 Hz. During eupnea the power spectra of the phrenic neurogram of both cats exhibited two prominent peaks, the first at 40–55 Hz and the second at approximately 100 Hz. The frequencies of these peaks correspond to those described in previous spectral analyses of the phrenic neurogram during eupnea where the lower-frequency peak has been described as medium-frequency oscillation (MFO) and the higher-frequency peak as high-frequency oscillation (HFO) [18,23]. In our results, the transition from eupnea to gasping was characterized by the loss of the MFO, and the appearance of a major peak in the 10–30 Hz range. This shift to a lower frequency during gasping contrasts with the finding of Richardson and Mitchell [22] where gasping resulted in a new spectral peak at a frequency higher than the eupneic HFO. The shift of power to a lower frequency during gasping, observed in our preliminary studies, suggests that there is a synchronization of neuronal firing at a frequency of 20–25 Hz during gasping. The maximal firing frequency of an individual neuron is presumably determined by the kinetics of the ion conductance changes associated with the action potential propagation, which require a finite time for activation and inactivation before a second action potential can be propagated. A frequency of 20–25 Hz is slower than the maximal frequency observed in individual phrenic motoneurons during eupnea (50 Hz), but may represent the maximum firing frequency of a respiratory neuron under the severe hypoxic conditions associated with gasping where channel conductance kinetics may be compromised [25].

When viewed in the time domain, the phrenic neurogram displays a characteristic "ramp" pattern during inspiration and decrementing activity during a short post-inspiratory period [6,12,13,15,26]. This pattern results from an orderly recruitment of phrenic premotor and motor units throughout the period of inspiration. Cohen et al. [27], observed both low- and high-frequency neurogram patterns in piglets at birth, but the high-frequency component was shown to increase with age [27]. They also claimed that high frequency oscillations arise from brain stem respiratory neurons in the medulla and the low-frequency component was not increased with age and was believed to originate from respiratory efferent systems. Later, Webber [28] showed in adult cats that both the early and late phases of the phrenic neurogram have a high frequency component, which is around 82 Hz. Only the late phase has a low frequency component, which is around 29 Hz.

We recently showed that the breathing activities for the young group are not periodic signals, and that the characteristics of phrenic neurograms rapidly change with respect to time [29]. Furthermore our results showed that the phrenic neurogram consists of several dominant burst type activities (circular structured components) corresponding to the early and late phases of the inspiratory activity. However, dominant burst type activities (circular structured components) were only present during the late phase of the phrenic neurogram when maturation proceeds. These results suggest that the phrenic neurogram is not a periodic signal and that its characteristics change rapidly during maturation. The dominant burst type activities disappeared during the early phase of the phrenic neurogram although the burst activity and the continuous activity remained, but both them appear at the late phase of the phrenic neurogram as maturation proceeds [29].

The objective of the study herein presented was to investigate the similarity on the time-frequency respiratory patters during eupnea and severe hypoxia (gasping) and to determine whether hypoxia results in changes in the time-frequency patterns of the respiratory motor output. We have examined the phrenic neurogram in both time and frequency domains during the first few weeks of postnatal life using time-frequency analysis methods to gain insight into the behavior of the respiratory neural network during eupnea and severe hypoxia.

## Methodology

### Experiment

Experiments were performed in decerebrate piglets of both sexes. Piglets were divided into three age groups: 3–6 days (n = 4), 10–15 days (n = 3) and 29–35 days (n = 3). The animals were anesthetized with 4% isoflurane in O_2_. The trachea was then cannulated for subsequent delivery of anesthesia (2–3% isoflurane in O_2_). Cannulation of the femoral artery and vein, peripheral chemodenervation, vagotomy, paralysis, and ventilation were performed. The scalp and underlying muscles were cut and the cerebral hemisphere and the diencephalon were removed. After exposing the mesencephalon, a mid-collicular cut was made and the remaining brain structures rostral to the incision were removed. After completion of the decerebration, anesthesia was removed. Piglets were chemically paralyzed for the rest of the experiment. A minimum of one hour was allowed to elapse between removal of anesthesia and data collection. Piglets were ventilated with 40% O_2 _in N_2 _during eupnea. Then, severe hypoxia was produced by inhalation of 3–5% O_2 _in N_2 _until gasping was observed in the phrenic neurogram. Phrenic neurogram activity was also recorded during 30 min of reoxygenation (40% O_2 _in N_2_).

Data was digitized on line by using a commercial data acquisition and analysis software program (ADI, Powerlab). The phrenic nerve was isolated in the neck at the level of C5 rootlet. The nerve was cut and placed on a bipolar electrode for neuronal recording. The raw phrenic neurogram was bandpass filtered (10 – 300 kHz) and sampled at 1 kHz [29].

### Continuous Wavelet Transform (CWT)

The continuous wavelet transform was utilized to analyze the phrenic neurogram signals. This transformation can be viewed as an inner product operation that allows one to measure the similarity or cross-correlation between the signal, s(t), and the wavelet function. The continuous wavelet transform of s(t) is defined as:



where **b **is a translation (shift) in time and **a **is the scale factor which represents a translation (shift) in frequency. In the study, we used the Morlet based CWT transform since it shows better time-frequency resolution compared to other orthogonal wavelet transform methods. The details of the Morlet based CWT are described elsewhere [30].

## Results

**Figure 1:**
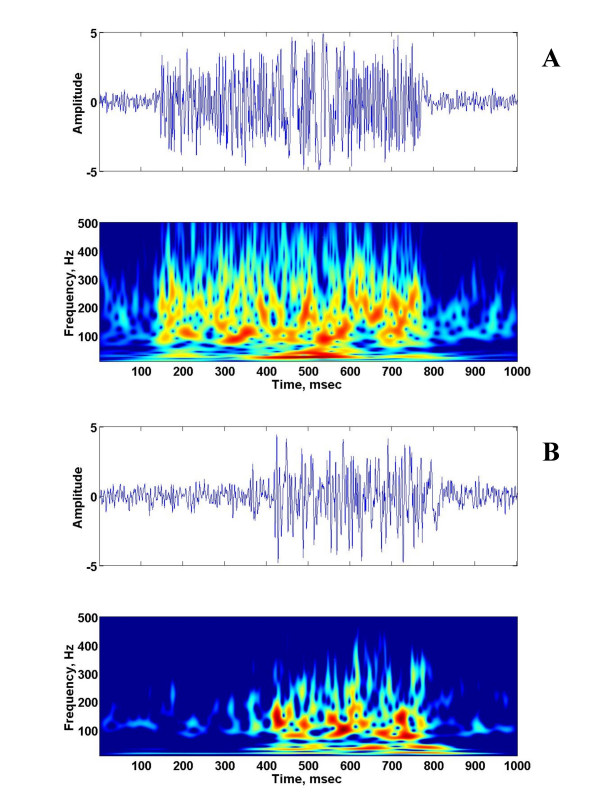
The raw phrenic neurogram and the corresponding time-frequency representation of the phrenic neurogram of a 3-day old piglet during eupnea (a) and gasping (b).

**Figure 2:**
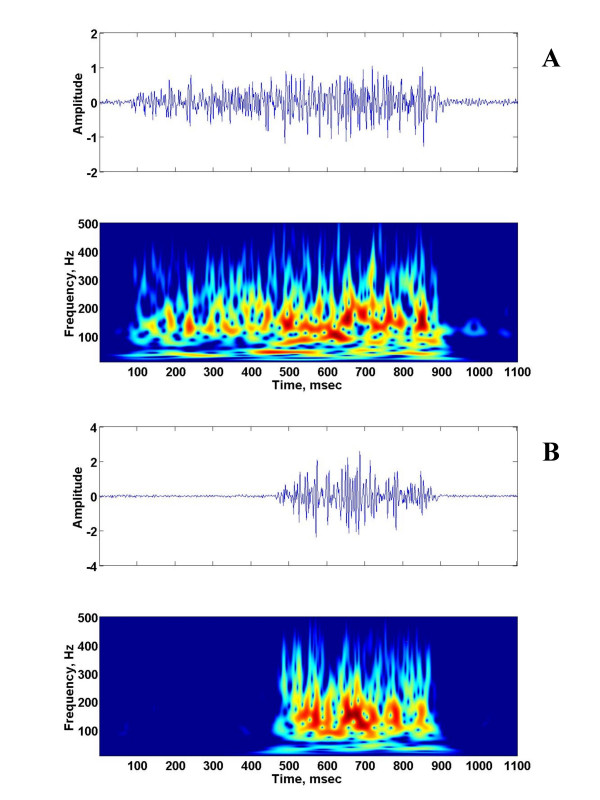
The raw phrenic neurogram and the corresponding time-frequency representation of the phrenic neurogram of a 10-day old piglet during eupnea (a) and gasping (b).

For each piglet, the time-frequency representations during eupnea and severe hypoxia were estimated and compared. Figures 1 and 2 show the raw and the corresponding time-frequency representation of the typical raw phrenic neurograms of a 3-day old piglet during eupnea and severe hypoxia, respectively. Although severe hypoxia (gasping) reduced the time duration of phrenic neurograms during inspiration and increased the expiratory duration, the time-frequency representations during early and late phases of phrenic neurogram during eupnea and gasping showed components between 30 and 300 Hz and demonstrated similarities. In addition, all 4 piglets in the young group exhibited gasping patterns when they were exposed to severe hypoxia.

**Figure 3:**
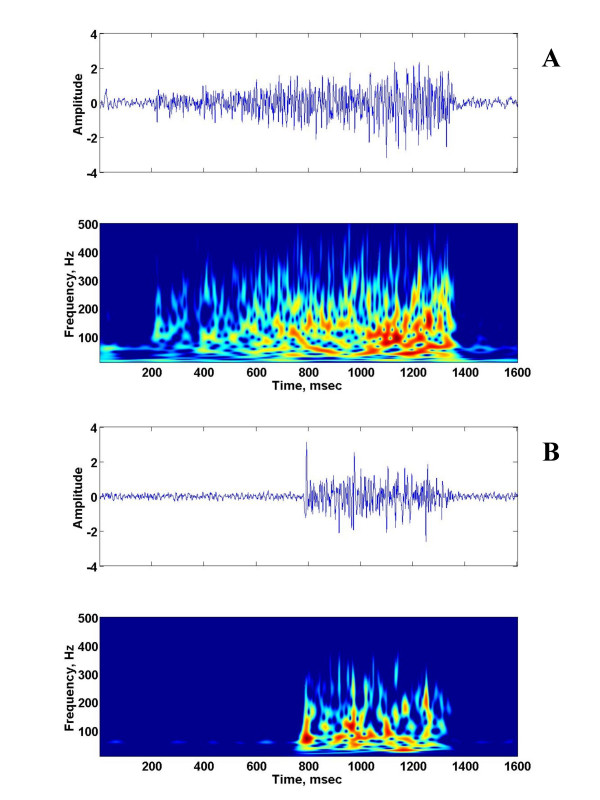
The raw phrenic neurogram and the corresponding time-frequency representation of the phrenic neurogram of a 30-day old piglet during eupnea (a) and gasping (b).

**Figure 4:**
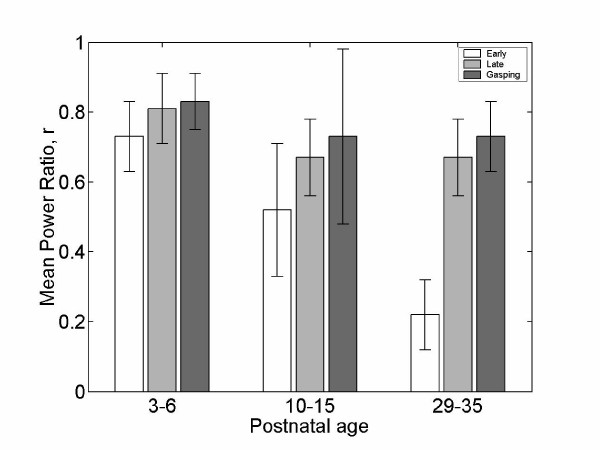
The mean ratio of the total energies above and below 150 Hz for the early and late phases of the phrenic neurogram during eupnea as well as the phrenic burst during gasping for 3 different age groups.

For the mid-age group, only one of 3 animals had gasping patterns and recovered when animals were reoxygenated. Figures 3 and 4 shows the similar features for a 10 days old piglet. The time frequency patterns were dominant between 30 and 300 Hz at the late phase of the phrenic neurogram during eupnea and about the same as those of gasping.

Figure 4 show the time-frequency patters for a 30-day old piglet. For the 29–35 days old age groups, the time frequency patterns between 30 and 300 Hz are only present for the late phase of the phrenic neurogram during eupnea. The time frequency patterns during gasping and the late phase of phrenic neurogram during eupnea showed considerable similarities. However, the patterns during the early phase of phrenic neurogram was not dominant and the signal components below 150 Hz were different from those marking phrenic neurograms during eupnea.

To investigate the similarity between the patterns in the early and late phases of the phrenic neurogram during eupnea and the patterns during gasping, time-frequency patterns for each piglet over 10 consecutive phrenic bursts during eupnea and 2–3 phrenic bursts during gasping were estimated for each group. Then, we calculated the mean total energies for four time-frequency regions, divided first in time (first and second half of the phrenic neurogram) and then frequency (above and below 150 Hz) during eupnea and the mean total energies below and above 150 Hz during gasping. The mean ratio of the total energies above and below 150 Hz for the early and late phases of the phrenic neurogram during eupnea as well as the phrenic burst during gasping were estimated. The mean ratios for the early, late phase during eupnea and gasping were 0.73 ± 0.1, 0.11 ± 0.1, 0.83 ± 0.19, respectively for the young group. They were 0.52 ± 0.17, 0.67 ± 0.1, 0.73 ± 0.25, for the mid-group and finally they were 0.22 ± 0.09, 0.67 ± 0.12, 0.73 ± 0.18, for the old age groups. Figure 7 summarizes the results. The mean ratios for the early and late phases of the phrenic neurograms during eupnea when compared to those of gasping were not statistically significant for the young age group. As maturation proceeds, the mean ratios for the early phase of phrenic neurograms during eupnea and phrenic bursts during gasping were statistically different although those for the late phases of phrenic bursts during eupnea and phrenic bursts during gasping remained statistically not different. Statistical analysis was performed via an analysis of variance (ANOVA) test.

## Discussion and conclusion

Our previous study based on time-frequency analysis methods showed that the time-frequency patterns at the early and late phases of the phrenic neurogram were the same for the 3–6 days old age group. As maturation proceeds, the early phase of the phrenic neurograms demonstrated patterns below 150 Hz that were not dominant, but the patterns for the last phase of phrenic neurograms remained the same and were not influenced by maturation. In this study, we estimated the time-frequency patterns during early and late phases of phrenic neurograms during eupnea and compared them with those of gasping in order to investigate the similarities between these patterns.

Our preliminary data indicated that the patterns during early and late phases of the phrenic neurogram during eupnea are similar to those during gasping for the 3–6 days old group.

The piglets in the young group were very resistive and showed strong responses during gasping in all 4 piglets in this study. However, the mid-group (10–15 days) failed to gasp in 2 of 3 animals. But, all three animals in the old group exhibited the gasping patterns like those in the 3–6 days old group. Therefore, we suggest that the animals in the mid-group could be more vulnerable compared to those in the young and old age groups. In addition, the patterns during early and late phases of phrenic neurogram were almost the same as those of gasping. As maturation proceeds, the similarity between the late phase of phrenic neurogram and gasping remained. Nevertheless, hypoxia significantly reduced the phrenic activities in the early phase of phrenic neurograms and caused a shift in the associated frequency components toward the lower frequency range (i.e., below 150 Hz). Hypoxia significantly increased the expiratory duration and reduced the inspiratory duration (especially, as maturation proceeds). Our most significant finding is that hypoxia silences the neural activity in the early phase of phrenic neurogram regardless of maturation.

Although we do not know the exact mechanism underlying these changes in the patterns of the phrenic neurograms from eupnea to gasping, we speculate that gasping silences phrenic neurons responsible for the neural activities in the early phase of the phrenic neurogram and does not influence phrenic neurons responsible for the neural activities in the late phase of the phrenic neurogram during inspiration. In addition, it also significantly increases the duration of the phrenic neurogram during expiration. We also noted that patterns observed during gasping did not change significantly as maturation proceeds. We speculate that severe hypoxia silences respiratory neurons responsible for both early and late phases of phrenic neurograms in 2 of 3 piglets in the mid-group. We suspect that a reduction in the number of dendrites per cell after 2 weeks of maturation could be responsible for the failure of gasping patterns in these piglets [31].
